# *rac*-Ethyl 4-hy­droxy-6-(2-hy­droxy­phen­yl)-2-oxo-4-(trifluoro­methyl)per­hydro­pyrimidine-5-carboxyl­ate

**DOI:** 10.1107/S1600536810021355

**Published:** 2010-06-16

**Authors:** Xiao-Ping Song, Gong-Chun Li, Feng-Xiang Zhu, Chang-Zeng Wu

**Affiliations:** a College of Chemistry and Chemical Engineering, Xuchang University, Xuchang, Henan Province 461000, People’s Republic of China

## Abstract

In the title compound, C_14_H_15_F_3_N_2_O_5_, prepared by reaction of 2-hy­droxy­benzaldehyde, ethyl 4,4,4-trifluoro-3-oxobutano­ate and urea, the tetra­pyrimidine ring adopts a half-chair conformation. The crystal structure is stabilized by five inter­molecular hydrogen bonds, three O—H⋯O and two N—H⋯O, giving cyclic dimers (through three hydrogen bonds) which are further extended into a two-dimensional network.

## Related literature

For the bioactivity of dihydro­pyrimidines, see: Brier *et al.* (2004 ▶); Cochran *et al.* (2005 ▶); Moran *et al.* (2007 ▶); Zorkun *et al.* (2006 ▶). For the bioactivity of organofluorine compounds, see: Hermann *et al.* (2003 ▶); Ulrich (2004 ▶).
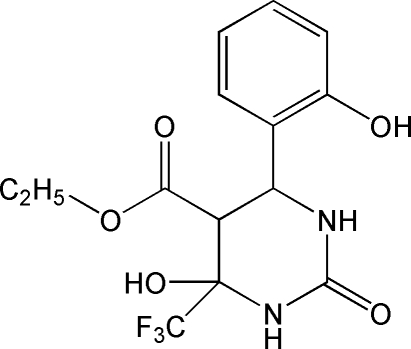


## Experimental

### 

#### Crystal data

C_14_H_15_F_3_N_2_O_5_
*M* *_r_* = 348.28
Monoclinic, 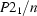

*a* = 12.0940 (15) Å
*b* = 8.665 (1) Å
*c* = 14.3110 (18) Å
β = 93.987 (6)°
*V* = 1496.1 (3) Å^3^
*Z* = 4
Mo *K*α radiation
μ = 0.14 mm^−1^
*T* = 113 K
0.26 × 0.22 × 0.20 mm

#### Data collection

Rigaku Saturn724 CCD diffractometer
Absorption correction: multi-scan (*CrystalClear*; Rigaku, 2009 ▶) *T*_min_ = 0.965, *T*_max_ = 0.973
15222 measured reflections
3558 independent reflections
2461 reflections with *I* > 2σ(*I*)’
*R*_int_ = 0.043

#### Refinement

*R*[*F* ^2^ > 2σ(*F* ^2^)] = 0.033
*wR*(*F* ^2^) = 0.075
*S* = 0.96
3558 reflections
234 parameters
H atoms treated by a mixture of independent and constrained refinement
Δρ_max_ = 0.29 e Å^−3^
Δρ_min_ = −0.17 e Å^−3^

### 

Data collection: *CrystalClear-SM Expert* (Rigaku, 2009 ▶); cell refinement: *CrystalClear-SM Expert*; data reduction: *CrystalClear-SM Expert*; program(s) used to solve structure: *SHELXS97* (Sheldrick, 2008 ▶); program(s) used to refine structure: *SHELXL97* (Sheldrick, 2008 ▶); molecular graphics: *CrystalStructure* (Rigaku, 2009 ▶); software used to prepare material for publication: *CrystalStructure*.

## Supplementary Material

Crystal structure: contains datablocks global, I. DOI: 10.1107/S1600536810021355/zs2042sup1.cif

Structure factors: contains datablocks I. DOI: 10.1107/S1600536810021355/zs2042Isup2.hkl

Additional supplementary materials:  crystallographic information; 3D view; checkCIF report

## Figures and Tables

**Table 1:** Hydrogen-bond geometry (Å, °)

*D*—H⋯*A*	*D*—H	H⋯*A*	*D*⋯*A*	*D*—H⋯*A*
N1—H4⋯O1^i^	0.836 (14)	2.244 (14)	3.0760 (13)	174.1 (14)
N2—H5⋯O2^ii^	0.867 (14)	2.040 (15)	2.9064 (14)	177.4 (13)
O1—H1⋯O5^i^	0.858 (17)	2.588 (16)	3.0983 (13)	119.2 (13)
O1—H1⋯O3^i^	0.858 (17)	2.013 (17)	2.8232 (13)	157.1 (15)
O5—H6⋯O2^iii^	0.912 (16)	1.759 (17)	2.6685 (12)	175.8 (14)

Symmetry codes: (i) 
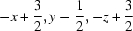
; (ii) 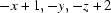; (iii) 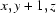.

**Fig. 1.**
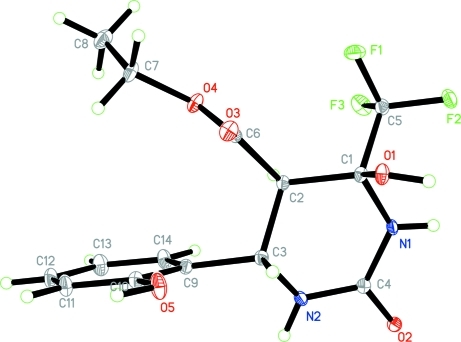
Molecular configuration and atom numbering scheme for (I), with displacement ellipsoids drawn at the 30% probability level.

**Fig. 2.**
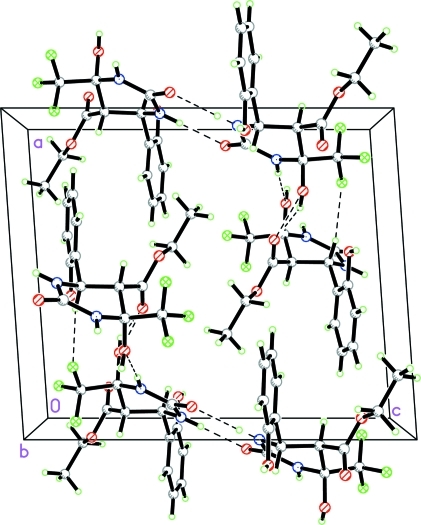
The packing diagram of the title compound. Intermolecular hydrogen bonds are shown as dashed line.

## supplementary crystallographic information

### Comment

Dihydropyrimidine (DHPM) derivatives can be used as potential calcium channel blockers (Zorkun *et al.*, 2006), inhibitors of mitotic kinesin Eg5 for treating cancer (Cochran *et al.*, 2005; Brier *et al.*, 2004) and as TRPA1 modulators for treating pain (Moran *et al.*, 2007). In addition, compounds that contain fluorine have special bioactivity, *e.g.* flumioxazin is a widely used herbicide (Hermann *et al.*, 2003; Ulrich, 2004). This led us to focus our attention on the synthesis and bioactivity of these important fused perfluoroalkylated heterocyclic compounds. During the synthesis of DHPM derivatives, the title compound, an intermediate C_14_H_15_F_3_N_2_O_5_ (I) was isolated and the structure confirmed by X-ray diffraction, in order to elucidate the reaction mechanism.

In the structure of the title molecule, the dihydropyrimidine ring adopts a half-chair conformation. The crystal structure is stabilized by five intermolecular hydrogen bonds, three O—H···O and two N—H···O (Table 1), giving cyclic dimers which are further extended into a two-dimensional network (Fig. 2).

dimension?

### Experimental

The title compound was synthesized refluxing for 3 h, a stirred solution of 2-hydroxybenzaldehyde (0.61 g, 5 mmol), ethyl 4,4,4-trifluoro-3-oxobutanoate (1.11 g, 6 mmol) and urea (0.45 g, 7.5 mmol) in 5 ml of ethanol, the reaction catalyzed by sulfamic acid (0.15 g). The solvent was evaporated *in vacuo* and the residue was washed with water. The title compound was recrystallized from 50% aqueous ethanol and single crystals of (I) were obtained by slow evaporation.

### Refinement

Hydrogen atoms involved in hydrogen-bonding inetractions were located by difference methods and their positional and isotropic displacement parameters were refined. Other H atoms were placed in calculated positions, with C—H(aromatic) = 0.95 Å and C—H(aliphatic) = 0.98 or 0.99 Å, and treated as riding, with *U*_iso_(H) = 1.2*U*eq(C).

### Crystal data

**Table:**

C_14_H_15_F_3_N_2_O_5_	*F*(000) = 720
*M**_r_* = 348.28	*D*_x_ = 1.546 Mg m^−^^3^
Monoclinic, *P*2_1_/*n*	Mo *K*α radiation, λ = 0.71075 Å
Hall symbol: -P 2yn	Cell parameters from 4600 reflections
*a* = 12.0940 (15) Å	θ = 1.7–28.0°
*b* = 8.665 (1) Å	µ = 0.14 mm^−^^1^
*c* = 14.3110 (18) Å	*T* = 113 K
β = 93.987 (6)°	Prism, colorless
*V* = 1496.1 (3) Å^3^	0.26 × 0.22 × 0.20 mm
*Z* = 4	

### Data collection

**Table:**

Rigaku Saturn724 CCD diffractometer	3558 independent reflections
Radiation source: rotating anode	2461 reflections with *I* > 2σ(*I*)'
multilayer	*R*_int_ = 0.043
Detector resolution: 14.222 pixels mm^-1^	θ_max_ = 27.9°, θ_min_ = 2.1°
ω scans	*h* = −15→15
Absorption correction: multi-scan (*CrystalClear*; Rigaku, 2009)	*k* = −10→11
*T*_min_ = 0.965, *T*_max_ = 0.973	*l* = −18→15
15222 measured reflections	

### Refinement

**Table:**

Refinement on *F*^2^	Primary atom site location: structure-invariant direct methods
Least-squares matrix: full	Secondary atom site location: difference Fourier map
*R*[*F*^2^ > 2σ(*F*^2^)] = 0.033	Hydrogen site location: inferred from neighbouring sites
*wR*(*F*^2^) = 0.075	H atoms treated by a mixture of independent and constrained refinement
*S* = 0.96	*w* = 1/[σ^2^(*F*_o_^2^) + (0.0366*P*)^2^] where *P* = (*F*_o_^2^ + 2*F*_c_^2^)/3
3558 reflections	(Δ/σ)_max_ < 0.001
234 parameters	Δρ_max_ = 0.29 e Å^−^^3^
0 restraints	Δρ_min_ = −0.17 e Å^−^^3^

### Special details

**Table:**

Geometry. All e.s.d.'s (except the e.s.d. in the dihedral angle between two l.s. planes) are estimated using the full covariance matrix. The cell e.s.d.'s are taken into account individually in the estimation of e.s.d.'s in distances, angles and torsion angles; correlations between e.s.d.'s in cell parameters are only used when they are defined by crystal symmetry. An approximate (isotropic) treatment of cell e.s.d.'s is used for estimating e.s.d.'s involving l.s. planes.
Refinement. Refinement of *F*^2^ against ALL reflections. The weighted *R*-factor *wR* and goodness of fit *S* are based on *F*^2^, conventional *R*-factors *R* are based on *F*, with *F* set to zero for negative *F*^2^. The threshold expression of *F*^2^ > σ(*F*^2^) is used only for calculating *R*-factors(gt) *etc*. and is not relevant to the choice of reflections for refinement. *R*-factors based on *F*^2^ are statistically about twice as large as those based on *F*, and *R*- factors based on ALL data will be even larger.

### Fractional atomic coordinates and isotropic or equivalent isotropic displacement parameters (Å^2^)

**Table:**

	*x*	*y*	*z*	*U*_iso_*/*U*_eq_	
F1	0.63046 (7)	0.13597 (8)	0.56068 (5)	0.0284 (2)	
F2	0.70104 (6)	−0.07799 (8)	0.61474 (5)	0.02243 (19)	
F3	0.52483 (6)	−0.04190 (9)	0.61064 (5)	0.0278 (2)	
O1	0.73825 (7)	0.17971 (10)	0.73408 (6)	0.0166 (2)	
O2	0.57743 (7)	−0.12700 (9)	0.92883 (6)	0.0155 (2)	
O3	0.61021 (7)	0.46053 (9)	0.68641 (6)	0.0188 (2)	
O4	0.44283 (7)	0.37553 (9)	0.63011 (6)	0.0189 (2)	
O5	0.57096 (8)	0.57378 (10)	0.88466 (7)	0.0226 (2)	
N1	0.62882 (9)	−0.02277 (12)	0.79262 (7)	0.0144 (2)	
N2	0.52802 (9)	0.11947 (11)	0.89483 (8)	0.0151 (2)	
C1	0.63604 (10)	0.10070 (14)	0.72522 (9)	0.0136 (3)	
C2	0.54011 (10)	0.21315 (13)	0.73746 (8)	0.0128 (3)	
H2	0.4693	0.1572	0.7201	0.015*	
C3	0.53875 (10)	0.26147 (13)	0.84121 (8)	0.0139 (3)	
H3	0.6103	0.3134	0.8614	0.017*	
C4	0.57828 (10)	−0.01283 (14)	0.87496 (9)	0.0137 (3)	
C5	0.62326 (10)	0.02811 (14)	0.62678 (9)	0.0169 (3)	
C6	0.53918 (10)	0.36240 (14)	0.68090 (8)	0.0144 (3)	
C7	0.41216 (11)	0.52976 (14)	0.59613 (10)	0.0219 (3)	
H7A	0.4704	0.5723	0.5582	0.026*	
H7B	0.4024	0.6003	0.6494	0.026*	
C8	0.30531 (11)	0.51220 (17)	0.53746 (10)	0.0273 (3)	
H8A	0.3166	0.4433	0.4847	0.033*	
H8B	0.2806	0.6135	0.5137	0.033*	
H8C	0.2489	0.4683	0.5757	0.033*	
C9	0.44302 (10)	0.37055 (13)	0.85540 (8)	0.0145 (3)	
C10	0.46361 (10)	0.52609 (14)	0.87614 (8)	0.0157 (3)	
C11	0.37451 (11)	0.62576 (15)	0.88661 (9)	0.0203 (3)	
H11	0.3882	0.7312	0.9014	0.024*	
C12	0.26690 (11)	0.57248 (15)	0.87573 (9)	0.0233 (3)	
H12	0.2071	0.6412	0.8836	0.028*	
C13	0.24560 (12)	0.41890 (15)	0.85339 (10)	0.0248 (3)	
H13	0.1715	0.3824	0.8452	0.030*	
C14	0.33378 (11)	0.31964 (15)	0.84326 (9)	0.0204 (3)	
H14	0.3194	0.2146	0.8277	0.024*	
H1	0.7889 (15)	0.1108 (18)	0.7422 (12)	0.046 (5)*	
H4	0.6684 (12)	−0.1011 (15)	0.7884 (9)	0.021 (4)*	
H5	0.4973 (12)	0.1248 (15)	0.9477 (11)	0.025 (4)*	
H6	0.5734 (13)	0.6771 (19)	0.8967 (11)	0.043 (5)*	

### Atomic displacement parameters (Å^2^)

**Table:**

	*U*^11^	*U*^22^	*U*^33^	*U*^12^	*U*^13^	*U*^23^
F1	0.0430 (5)	0.0269 (4)	0.0157 (4)	0.0048 (4)	0.0052 (4)	0.0046 (3)
F2	0.0214 (4)	0.0232 (4)	0.0232 (4)	0.0060 (3)	0.0047 (3)	−0.0049 (3)
F3	0.0180 (4)	0.0365 (5)	0.0285 (5)	−0.0054 (4)	−0.0001 (3)	−0.0131 (4)
O1	0.0097 (4)	0.0138 (5)	0.0264 (5)	−0.0005 (4)	0.0010 (4)	0.0003 (4)
O2	0.0196 (5)	0.0110 (4)	0.0161 (5)	0.0002 (4)	0.0029 (4)	0.0015 (4)
O3	0.0161 (5)	0.0168 (5)	0.0234 (5)	−0.0030 (4)	−0.0004 (4)	0.0028 (4)
O4	0.0151 (5)	0.0168 (5)	0.0241 (5)	0.0006 (4)	−0.0041 (4)	0.0051 (4)
O5	0.0165 (5)	0.0134 (5)	0.0381 (6)	−0.0009 (4)	0.0021 (4)	−0.0064 (4)
N1	0.0154 (5)	0.0115 (5)	0.0168 (6)	0.0045 (4)	0.0049 (4)	0.0010 (4)
N2	0.0197 (6)	0.0118 (5)	0.0146 (6)	0.0027 (4)	0.0065 (4)	0.0009 (4)
C1	0.0105 (6)	0.0133 (6)	0.0172 (7)	−0.0007 (5)	0.0014 (5)	0.0020 (5)
C2	0.0114 (6)	0.0120 (6)	0.0150 (7)	0.0009 (5)	0.0013 (5)	0.0010 (5)
C3	0.0135 (6)	0.0131 (6)	0.0152 (7)	0.0001 (5)	0.0017 (5)	0.0014 (5)
C4	0.0113 (6)	0.0132 (6)	0.0163 (7)	−0.0022 (5)	−0.0009 (5)	−0.0015 (5)
C5	0.0148 (6)	0.0171 (6)	0.0189 (7)	0.0016 (5)	0.0027 (5)	0.0010 (5)
C6	0.0128 (6)	0.0166 (7)	0.0138 (7)	0.0021 (5)	0.0020 (5)	−0.0009 (5)
C7	0.0209 (7)	0.0161 (7)	0.0282 (8)	0.0041 (5)	−0.0009 (6)	0.0072 (6)
C8	0.0217 (7)	0.0306 (8)	0.0291 (8)	0.0048 (6)	−0.0013 (6)	0.0094 (6)
C9	0.0148 (6)	0.0139 (6)	0.0153 (7)	0.0023 (5)	0.0036 (5)	0.0024 (5)
C10	0.0149 (6)	0.0150 (6)	0.0172 (7)	0.0009 (5)	0.0015 (5)	0.0004 (5)
C11	0.0209 (7)	0.0143 (7)	0.0257 (8)	0.0033 (5)	0.0018 (6)	−0.0028 (6)
C12	0.0178 (7)	0.0223 (7)	0.0302 (8)	0.0081 (6)	0.0045 (6)	−0.0003 (6)
C13	0.0150 (7)	0.0246 (7)	0.0353 (8)	0.0003 (6)	0.0049 (6)	−0.0016 (6)
C14	0.0183 (7)	0.0147 (7)	0.0289 (8)	−0.0005 (5)	0.0059 (6)	−0.0017 (6)

### Geometric parameters (Å, °)

**Table:**

F1—C5	1.3370 (14)	C2—C3	1.5438 (17)
F2—C5	1.3350 (14)	C2—H2	1.0000
F3—C5	1.3418 (14)	C3—C9	1.5191 (16)
O1—C1	1.4110 (14)	C3—H3	1.0000
O1—H1	0.858 (17)	C7—C8	1.4990 (19)
O2—C4	1.2547 (14)	C7—H7A	0.9900
O3—C6	1.2073 (14)	C7—H7B	0.9900
O4—C6	1.3347 (15)	C8—H8A	0.9800
O4—C7	1.4611 (14)	C8—H8B	0.9800
O5—C10	1.3599 (15)	C8—H8C	0.9800
O5—H6	0.912 (16)	C9—C14	1.3922 (17)
N1—C4	1.3674 (15)	C9—C10	1.3986 (17)
N1—C1	1.4472 (16)	C10—C11	1.3971 (17)
N1—H4	0.836 (14)	C11—C12	1.3795 (18)
N2—C4	1.3373 (16)	C11—H11	0.9500
N2—C3	1.4606 (15)	C12—C13	1.3886 (19)
N2—H5	0.867 (14)	C12—H12	0.9500
C1—C2	1.5344 (16)	C13—C14	1.3856 (18)
C1—C5	1.5407 (18)	C13—H13	0.9500
C2—C6	1.5254 (16)	C14—H14	0.9500
			
C1—O1—H1	106.7 (11)	F3—C5—C1	111.93 (10)
C6—O4—C7	116.81 (10)	O3—C6—O4	124.46 (11)
C10—O5—H6	109.5 (10)	O3—C6—C2	125.69 (12)
C4—N1—C1	125.39 (10)	O4—C6—C2	109.59 (10)
C4—N1—H4	113.9 (9)	O4—C7—C8	106.41 (10)
C1—N1—H4	119.4 (9)	O4—C7—H7A	110.4
C4—N2—C3	123.51 (10)	C8—C7—H7A	110.4
C4—N2—H5	117.4 (9)	O4—C7—H7B	110.4
C3—N2—H5	118.2 (9)	C8—C7—H7B	110.4
O1—C1—N1	113.07 (10)	H7A—C7—H7B	108.6
O1—C1—C2	110.26 (10)	C7—C8—H8A	109.5
N1—C1—C2	108.19 (9)	C7—C8—H8B	109.5
O1—C1—C5	108.17 (9)	H8A—C8—H8B	109.5
N1—C1—C5	107.50 (10)	C7—C8—H8C	109.5
C2—C1—C5	109.58 (10)	H8A—C8—H8C	109.5
C6—C2—C1	117.10 (9)	H8B—C8—H8C	109.5
C6—C2—C3	106.28 (10)	C14—C9—C10	119.03 (11)
C1—C2—C3	109.92 (10)	C14—C9—C3	120.77 (11)
C6—C2—H2	107.7	C10—C9—C3	120.12 (11)
C1—C2—H2	107.7	O5—C10—C11	122.76 (11)
C3—C2—H2	107.7	O5—C10—C9	117.82 (10)
N2—C3—C9	110.95 (9)	C11—C10—C9	119.42 (11)
N2—C3—C2	106.50 (10)	C12—C11—C10	120.64 (12)
C9—C3—C2	110.89 (10)	C12—C11—H11	119.7
N2—C3—H3	109.5	C10—C11—H11	119.7
C9—C3—H3	109.5	C11—C12—C13	120.35 (12)
C2—C3—H3	109.5	C11—C12—H12	119.8
O2—C4—N2	121.47 (11)	C13—C12—H12	119.8
O2—C4—N1	120.20 (11)	C14—C13—C12	119.15 (13)
N2—C4—N1	118.31 (11)	C14—C13—H13	120.4
F2—C5—F1	107.99 (10)	C12—C13—H13	120.4
F2—C5—F3	106.92 (10)	C13—C14—C9	121.39 (12)
F1—C5—F3	107.15 (10)	C13—C14—H14	119.3
F2—C5—C1	111.86 (10)	C9—C14—H14	119.3
F1—C5—C1	110.74 (10)		
			
C4—N1—C1—O1	98.15 (14)	O1—C1—C5—F3	−176.35 (10)
C4—N1—C1—C2	−24.26 (16)	N1—C1—C5—F3	61.24 (13)
C4—N1—C1—C5	−142.52 (11)	C2—C1—C5—F3	−56.12 (13)
O1—C1—C2—C6	49.05 (14)	C7—O4—C6—O3	−13.82 (17)
N1—C1—C2—C6	173.17 (10)	C7—O4—C6—C2	160.62 (10)
C5—C1—C2—C6	−69.90 (13)	C1—C2—C6—O3	−60.93 (16)
O1—C1—C2—C3	−72.30 (12)	C3—C2—C6—O3	62.31 (15)
N1—C1—C2—C3	51.82 (13)	C1—C2—C6—O4	124.72 (11)
C5—C1—C2—C3	168.75 (9)	C3—C2—C6—O4	−112.04 (11)
C4—N2—C3—C9	158.86 (11)	C6—O4—C7—C8	176.95 (10)
C4—N2—C3—C2	38.09 (16)	N2—C3—C9—C14	−51.23 (16)
C6—C2—C3—N2	174.26 (10)	C2—C3—C9—C14	66.90 (14)
C1—C2—C3—N2	−58.11 (12)	N2—C3—C9—C10	132.13 (12)
C6—C2—C3—C9	53.45 (12)	C2—C3—C9—C10	−109.73 (13)
C1—C2—C3—C9	−178.92 (9)	C14—C9—C10—O5	−177.92 (11)
C3—N2—C4—O2	171.65 (11)	C3—C9—C10—O5	−1.22 (17)
C3—N2—C4—N1	−10.02 (18)	C14—C9—C10—C11	1.67 (18)
C1—N1—C4—O2	−179.52 (11)	C3—C9—C10—C11	178.36 (11)
C1—N1—C4—N2	2.12 (18)	O5—C10—C11—C12	178.89 (12)
O1—C1—C5—F2	63.67 (12)	C9—C10—C11—C12	−0.68 (19)
N1—C1—C5—F2	−58.74 (13)	C10—C11—C12—C13	−0.5 (2)
C2—C1—C5—F2	−176.10 (9)	C11—C12—C13—C14	0.7 (2)
O1—C1—C5—F1	−56.85 (13)	C12—C13—C14—C9	0.3 (2)
N1—C1—C5—F1	−179.26 (10)	C10—C9—C14—C13	−1.49 (19)
C2—C1—C5—F1	63.38 (12)	C3—C9—C14—C13	−178.16 (12)

### Hydrogen-bond geometry (Å, °)

**Table:**

*D*—H···*A*	*D*—H	H···*A*	*D*···*A*	*D*—H···*A*
N1—H4···O1^i^	0.836 (14)	2.244 (14)	3.0760 (13)	174.1 (14)
N2—H5···O2^ii^	0.867 (14)	2.040 (15)	2.9064 (14)	177.4 (13)
O1—H1···O5^i^	0.858 (17)	2.588 (16)	3.0983 (13)	119.2 (13)
O1—H1···O3^i^	0.858 (17)	2.013 (17)	2.8232 (13)	157.1 (15)
O5—H6···O2^iii^	0.912 (16)	1.759 (17)	2.6685 (12)	175.8 (14)

Symmetry codes: (i) −*x*+3/2, *y*−1/2, −*z*+3/2; (ii) −*x*+1, −*y*, −*z*+2; (iii) *x*, *y*+1, *z*.
